# An outcome analysis of self-expandable metallic stents in central airway obstruction: a cohort study

**DOI:** 10.1186/1749-8090-6-46

**Published:** 2011-04-08

**Authors:** Fu-Tsai Chung, Hao-Cheng Chen, Chun-Liang Chou, Chih-Teng Yu, Chih-Hsi Kuo, Han-Pin Kuo, Shu-Min Lin

**Affiliations:** 1 Department of Thoracic Medicine, Chang Gung Memorial Hospital, Chang Gung University, College of Medicine, No. 199 Tun Hwa N. Rd., Taipei City 10507, Taiwan; 2 Graduate Institute of Clinical Medical Sciences, College of Medicine, Chang Gung University, No. 259, Wun Hua 1st Rd., Gueishan Township, Taoyuan County 333, Taiwan

## Abstract

**Background:**

Self-expandable metallic stents (SEMSs) have provided satisfactory management of central airway obstruction. However, the long-term benefits and complications of this management modality in patients with benign and malignant obstructing lesions after SEMS placement are unclear. We performed this cohort study to analyze the outcomes of Ultraflex SEMSs in patients with tracheobronchial diseases.

**Methods:**

Of 149 patients, 72 with benign and 77 with malignant tracheobronchial disease received 211 SEMSs (benign, 116; malignant, 95) and were retrospectively reviewed in a tertiary hospital.

**Results:**

The baseline characteristics of patients who received SEMS implantation for benign conditions and those who underwent implantation for malignant conditions were significantly different. These characteristics included age (mean, 63.9 vs. 58; *p *< 0.01), gender (male, 62% vs. 90%; *p *< 0.0001), smoking (47% vs. 85%; *p *< 0.0001), forced expiratory volume in 1 second (mean, 0.9 vs. 1.47 L/s; *p *< 0.0001), follow-up days after SEMS implantation (median; 429 vs. 57; *p *< 0.0001), and use of covered SEMS (36.2% vs. 94.7%; *p *< 0.0001). Symptoms improved more after SEMS implantation in patients with benign conditions than in those with malignant conditions (76.7% vs. 51.6%; *p *< 0.0001). The overall complication rate after SEMS implantation in patients with benign conditions was higher than that in patients with malignancy (42.2% vs. 21.1%; *p *= 0.001). Successful management of SEMS migration, granulation tissue formation, and SEMS fracture occurred in 100%, 81.25%, and 85% of patients, respectively.

**Conclusions:**

Patients who received SEMS implantation owing to benign conditions had worse lung function and were older than those who received SEMS for malignancies. There was higher complication rate in patients with benign conditions after a longer follow-up period owing to the nature of the underlying diseases.

## Introduction

Patients with symptomatic central airway lesions can be treated with surgery or endoscopic intervention [1-3]. Owing to advances in endobronchial stents and insertion techniques, interventional bronchoscopic procedures have been widely used in patients with benign and malignant lesions [4-7]. Rigid and flexible bronchoscopies are the most common methods of stent implantation in these patients. Some patients are not candidates for surgical intervention or rigid bronchoscopy with a general anaesthetic, however, because of illness severity and comorbidities. Self-expandable metallic stents (SEMSs) can be successfully implanted with a flexible bronchoscope while the patient receives conscious sedation and a local anaesthetic [8-10]. Patients who are ineligible for surgical procedures or rigid bronchoscopy may undergo SEMS implantation to relieve their symptoms [11].

Although SEMSs provide satisfactory management of central airway obstruction in large tracheobronchial diseases, they are accompanied by complications such as migration, granulation tissue formation, impaired mucociliary clearance, recurrent lumen obstruction of the stent, and increased bacterial colonization [12-14]. Because of potentially hazardous complications, the US Food and Drug Administration (FDA) has warned that SEMS implantation should be considered only in patients who are ineligible for surgery, rigid bronchoscopy, or silicone stent implantation. However, previous studies [10,15] have concluded that SEMSs are useful management options for central airway obstruction in patients with benign and malignant diseases. The benefits and complications of SEMS placement in patients with benign obstructing lesions are unclear in the available medical literature.

This study was designed to investigate the long-term outcomes of patients with airway obstruction who received SEMSs. Most of the patients received an SEMS before the FDA warning about the placement of SEMSs in patients with benign airway obstruction. The goal of this study was to analyze the baseline characteristics, clinical features, overall symptomatic response, and complication rate after SEMS placement in patients with benign and malignant central airway obstructions. The management of SEMS-related complications was also investigated.

## Patients and Methods

### Design

This investigation was a retrospective study. Informed consents were obtained from all patients or their surrogates before bronchoscopic SEMS implantation and follow-up. Methodology and patient confidentiality were approved by our institutional review board (IRB). The IRB was also asked to review the design of the project in December 2006, and it approved this retrospective study in March 2007 (IRB No.: 98-3287B). The IRB confirmed that this study constituted an audit, which did not require patient consents.

### Patients

From August 2001 to March 2007, 149 patients (mean age ± standard deviation, 62.1 ± 15.4; range, 23-91) underwent 211 endoscopic airway stent placements at Chang Gung Memorial Hospital, a university-affiliated hospital in Taiwan. In total, 116 stents were used in 72 patients with benign tracheobronchial disease and 95 stents were used in 77 patients with malignancy. Thoracic surgeons were routinely consulted for the feasibility of surgical intervention or rigid bronchoscopy in all patients before SEMS implantation. If patients were unsuitable for surgical intervention owing to poor lung function, co-morbidities, or refusal to undergo surgery, SEMS implantation with fibre-optic bronchoscopy was used if other treatment options were unavailable. The development of new or progressive symptoms was closely monitored, and follow-up radiographic and bronchoscopic examinations were arranged.

### Stent implantation

Ultraflex (Boston Scientific, Natick, MA), a tightly woven, self-expandable metallic stent composed entirely of a single strand of nickel-titanium alloy, was the stent of choice for this study. Central airway stenosis was evaluated using chest computed tomography (CT) and bronchoscopy [16-18]. The principles of SEMS implantation in our institution under conscious sedation and local anaesthesia and the assessment of stent condition have been reported in previous studies [16,17]. The choice of stent length and type (with or without cover) was made according to previous endoscopic examination and chest CT scan. SEMSs were implanted at the choke point determined using a flow-volume curve, endobronchial ultrasonography, bronchoscopy, or three-dimensional CT before and after stenting [18,19].

Stents types (length, diameter, and covered or uncovered) were selected according to CT scan, bronchoscopic image, and physician choice. Covered stents were usually used in patients with malignant diseases to cover the tumour mass in the airway. Only 5 patients with malignancy received uncovered stents because of the critical location of the implanted stent. Tumours of the main bronchi near the main carina caused narrowing of the main bronchial orifices. Covered stent placement could reopen obstructed main bronchial orifices but risked covering the other main bronchial orifice. Uncovered stents were usually selected in patients with benign diseases for feasibility of stent removal, especially in the bronchial airway.

### Assessment of stent condition

A follow-up bronchoscopy was performed 48 hours after stent placement. The presence of incomplete stent expansion or an incomplete stented airway lumen was recorded so that post-procedure factors could be evaluated in follow-up bronchoscopic studies. In addition, each patient underwent bronchoscopic examination 1 week after implantation and every 3-6 months thereafter to evaluate stent position and degradation, granulation tissue formation, and airway alignment. If new or progressive symptoms including dyspnoea, severe cough, increased mucous production, or other symptoms that suggested stent fracture occurred, additional bronchoscopy was performed.

### Definition of SEMS complications

All possible complications related to SEMS placement were confirmed with bronchoscopic examination. According to patients' records, complications included stent migration, granulation tissue formation, stent fracture, and pneumothorax. SEMS fracture was defined as physical breakage [16,17].

Successful management was defined as the relief of complications without the need for additional procedures during the follow-up period. A total of 5 patients with SEMS migration lacked significant symptoms and required no further management. These patients were considered successfully managed but were included in the analysis of stent migration.

### Statistical analysis

Data are expressed as either group percentages (categorical variables) or mean ± SD (continuous variables). Time variables are expressed as median and interquartile range (IQR). Data were compared between patients with benign and patients with malignant conditions. Categorical variables were compared using the chi square or Fisher's exact test. Unpaired t-tests were used to compare continuous variables. The significant difference between the 2 groups was defined as a *p *value less than 0.05. All analyses were performed using SPSS software v. 10.0 (SPSS, Chicago, IL).

## Results

From August 2001 to March 2007, 149 patients (mean age ± standard deviation, 61.2 ± 15.7 years; range, 23-91) with benign (n = 72) and malignant (n = 77) tracheobronchial disease received 211 Ultraflex SEMSs (116 for benign conditions and 95 for malignant condition). The indications for SEMS implantation are listed in Table 1.

**Table 1:** Indications for SEMS placement

Conditions	Number of patients	Number of SEMSs
Malignant diseases		
Primary lung cancer with airway invasion		
NSCLC	35(45.4)	45(47.3)
SCLC	7(9.1)	7(7.4)
Carcinoid	1(1.3)	2(2.1)
Sacrcoma	1(1.3)	1(1.1)
Oesophageal cancer		
TE fistula	24(31.2)	31(32.6)
Tracheal stenosis for tumour invasion	3(3.9)	3(3.2)
Other malignancy with trachea invasion	2(2.6)	2(2.6)
Mediastinal mass compression	4(5.2)	4(5.2)
Subtotal	77	95
Benign diseases		
Malacia	29(40.3)	64(55.2)
Post-intubation stenosis	9(12.5)	11(9.5)
Post-TB stenosis	9(12.5)	11(9.5)
Granulation restenosis	9(12.5)	13(11.2)
Stent fracture	6(8.3)	6(5.2)
Goiter	4(5.6)	4(3.4)
Corrosive injury	2(2.8)	3(2.6)
Mediastinitis	2(2.8)	2(1.7)
Tracheitis	2(2.8)	2(1.7)
Subtotal	72	116
Total	149	211

Abbreviations: NSCLC, non-small cell lung cancer; SCLC, small cell lung cancer; SEMS, self-expandable metallic stent; TB, tuberculosis; TE, tracheo-oesophageal

The demographics of patients who underwent SEMS placement, including those with benign and malignant disease, are listed in Table 2. Patient characteristics between the benign and malignant airway obstruction groups were significantly different. These characteristics included age (63.9 ± 15.6 years vs. 58 ± 12.2 years; *p *= 0.006), gender (male, 62.1% vs. 90.5%; *p <*0.0001), smoking (47.4% vs. 85.3%; *p <*0.0001), forced expiratory volume in 1 second 0.9 ± 0.4 L/s vs. 1.47 ± 0.68 L/s; *p *< 0.0001), follow-up days after SEMS placement (median (IQR); 429 (141-856) vs. 57 (19-103); *p *< 0.0001), and use of covered SEMS (36.2% vs. 94.7%; *p <*0.0001). The clinical presentation was significantly different in patients with benign airway obstruction when compared with patients with malignant disease. This presentation included dyspnoea (95.7% vs. 48.4%; *p <*0.0001), cough (1.7% vs. 21.1%; *p <*0.0001), respiratory failure (2.6% vs. 17.9%; *p *= 0.0002), pneumonia (0% vs. 9.5%; *p *= 0.0007) and haemoptysis (0% vs. 3.2%; *p *= 0.05).

**Table 2:** Demographics of patients receiving SEMS placement

	Total (n = 211)	Benign (n = 116)	Malignant (n = 95)	*p *value¶
**Demography**				
Age (yrs)	61.2 ± 15.7	63.9 ± 15.6	58.0 ± 15.2	0.006
Gender, Male, n(%)	158(74.9)	72(66.1%)	86(90.5)	<. 0001
Smoking, n(%)	136(64.5)	55(47.4)	81(82.3)	<. 0001
FEV1(L/s)	1.05 ± 0.55	0.90 ± 0.40	1.47 ± 0.68	<. 0001
SEMS follow up days, median(IQR)	130(39-550)	429(141-856)	57(19-103)	<. 0001
Cover SEMS, n(%)	132(62.6)	42(36.2)	90(94.7)	<. 0001
**Clinical manifestation before SEMS implantation**				
Dyspnoea, n(%)	157(74.4)	111(95.7)	46(48.4)	<. 0001
Cough, n(%)	22(10.4)	2(1.7)	20(21.1)	<. 0001
Respiratory failure, n(%)	20(9.5)	3(2.6)	17(17.9)	.0002
Pneumonia, n(%)	9(4.3)	0(0)	9(9.5)	.0007
Haemoptysis, n(%)	3(1.4)	0(0)	3(3.2)	0.05

Abbreviations: FEV1, forced expiratory flow in 1 second; FVC, forced vital capacity; IQR, interquartile range; *p *value, benign group vs. malignant group; SEMS, self-expandable metallic stent

The clinical responses and complications after SEMS placement in patients with benign airway obstruction and malignant disease are listed in Table 3. Patients with benign airway obstruction had a clinical response after SEMS placement that was significantly better than that of patients with malignant disease (76.7% vs. 51.6%; *p *< 0.0001). The overall complication rate (42.2% vs. 21.1%; *p = *0.001) after SEMS implantation was higher in patients with benign conditions than in patients with malignancy. The 30-day complication rate (4.3% vs. 9.5%; *p *= 0.13) and the 60-day complication rate (8.6% vs. 15.8%; *p *= 0.11) related to SEMS placement were similar in both groups. Complications in patients with malignant airway obstruction presented earlier than those in patients with benign conditions (median, IQR; 211, 52-686 days vs. 33, 19-35 days; *p *= 0.0002).

**Table 3:** Summary of responses after SEMS implantation

Response	Total (n = 211)	Benign (n = 116)	Malignant (n = 95)	*p *value¶
Resolution of symptom, n(%)	138(65.4)	89(76.7)	49(51.6)	<. 0001
Complication related to SEMS in 30 days, n(%)	14(6.6)	5(4.3)	9(9.5)	0.134
Complication related to SEMS in 60 days, n(%)	25(11.8)	10(8.6)	15(15.8)	0.109
Overall complications related to SEMS, n(%)	69(32.7)	49(42.2)	20(21.1)	.0011
Time to complications developed, median (IQR)	87(33-435)	211(52-686)	33(15-59)	.0002
Complication episode per patient per month	0.006	0.008	0.01	-

Abbreviations: IQR, Interquartile range; *p *value, benign group vs. malignant group; SEMS, self-expandable metallic stent

Table 4 summarizes the complication rates and the time to detect complications after SEMS implantation. The complication rates after SEMS implantation, including stent migration (6.9% vs. 8.4%; *p *= 0.68), granulation tissue formation (19% vs. 10.5%; *p *= 0.09), and pneumothorax (0% vs. 1.1%; *p = *0.27), were similar in patients with benign conditions and those with malignant conditions. The fracture of SEMSs was significantly more frequent in patients with benign airway obstruction than in patients with malignant disease (16.4% vs. 1.1%; *p *< 0.0001). The time to detect granulation tissue formation after SEMS implantation in patients with benign airway obstruction was longer than that in patients with malignant conditions (median, IQR; 212, 59-489 days vs. 31, 19-35 days; *p = *0.005). The time to detect SEMS migration was similar between the 2 groups.

**Table 4:** Complication rates and time to detect complications after SEMS placement

	Total (n = 211)	Benign (n = 116)	Malignant (n = 95)	*p *value¶
**Complication rate**				
Stent migration, n(%)	16(7.6)	8(6.9)	8(8.4)	0.677
Granulation tissue formation, n(%)	32(15.2)	22(19.0)	10(10.5)	0.089
Stent fracture, n(%)	20(9.5)	19(16.4)	1(1.1)	0.0002
Pneumothorax, n(%)	1(0.5)	0(0)	1(1.1)	0.268
**Time to detect complication after SEMS implantation (days)**				
Stent migration, n(%)	18(8-55)	45(9-109)	16(3-32)	0.112
Granulation tissue formation, n(%)	75(33-378)	212(59-489)	31(19-35)	0.005
Stent fracture, n(%)	652(306-814)	686(277-856)	515	-
Pneumothorax, n(%)	2	none	2	-

Abbreviations: --, unable to compare *p *value; *p *value, benign group vs. malignant group; SEMS, self-expandable metallic stent

Table 5 summarizes the complications and resolution of symptoms after SEMS placement in patients with benign disease who underwent covered stent placement and those who underwent uncovered stent placement. The incidence of complications, including stent migration (9.5% vs. 5.4%; *p *= 0.458), granulation tissue formation (16.7% vs. 20.3%; *p *= 0.634), and stent fracture (11.9% vs. 18.9%; *p = *0.44), was similar in both groups. Resolution of symptoms after covered and uncovered SEMS placement in patients with benign disease was also similar (71.4% vs. 79.7%; *p *= 0.363).

**Table 5:** Complications and resolution of symptoms in patients with benign diseases after covered and uncovered stent placement

	Total (n = 116)	Covered (n = 42)	Uncovered (n = 74)	*p *value¶
**Complication rate**				
Stent migration, n(%)	8(6.9)	4(9.5)	4(5.4)	0.458
Granulation tissue formation, n(%)	22(19.0)	7 (16.7)	15(20.3)	0.634
Stent fracture, n(%)	19(16.4)	5(11.9)	14(18.9)	0.44
**Resolution of symptoms, n(%)**	89(76.7)	30 (71.4)	59 (79.7)	0.363

Abbreviations: *p *value, covered vs. uncovered stents; SEMS, self-expandable metallic stent

The management and outcome of complications after SEMS placement are listed in Table 6. In patients with stent migration, observation (n = 5, 32.2%), reposition (n = 4, 25%), placement of another stent (n = 3, 18.8%), and stent removal (n = 4, 25%) were used to manage this complication. Granulation tissue formation related to SEMS placement (n = 32) was managed with electrocautery (n = 12, 37.5%), balloon dilatation (n = 1, 3.1%), stent removal (n = 12, 37.5%), or implantation of another stent (n = 7, 21.9%). Stent fracture (n = 20) was managed with removal of the fractured stent (n = 10, 50%) or implantation of another stent (n = 5, 25%). In total, 5 fractured stents (25%) resolved without intervention owing to minimal protrusion of the stent with patent lumen and preserved architecture. Successful management of SEMS migration, granulation tissue formation, and SEMS fracture occurred in 100%, 81.25%, and 85% of patients, respectively.

**Table 6:** Management and outcomes of SEMS-related complications

Complication	Management	Total, n(%)	Successful management, n(%)
**Migration**			
	None	5(32.2%)	5(32.5%)
	Reposition	4(25%)	4(25%)
	Another SEMS stenting	3(18.8%)	3(18.8%)
	SEMS removal	4(25%)	4(25%)
	Subtotal	16(100%)	16(100%)
**Granulation tissue formation**			
	Electrocautery only	12(37.5%)	9(28.1%)
	Balloon dilatation	1(3.1%)	1(3.1%)
	SEMS removal	12(37.5%)	11(34.5%)
	Another SEMS stenting	7(21.9%)	5(15.6%)
	Subtotal	32(100%)	26(81.25%)
**Stent fracture**			
	None	5(25%)	5(25%)
	SEMS removal	10(50%)	9(45%)
	Another SEMS stenting	5(25%)	3(15%)
	Subtotal	20(100%)	17(85%)

Abbreviation: SEMS, self-expandable metallic stent

## Discussion

In patients with central airway obstruction that is not amenable to surgery or that is medically inoperable, airway stenting may be the only possible treatment [20]. Silicone stents remain the first choice in benign airway obstruction except in patients with airway wall malacia or distal/angular stenosis. In these patients, SEMSs are generally indicated [21,22]. SEMSs have been widely used in benign and malignant airway obstruction and can be successfully implanted with a flexible bronchoscope with conscious sedation and local anaesthesia [9,10]. Unlike silicone stents, SEMSs have advantages such as lower migration rate, greater cross-sectional airway diameter owing to thinner wall construction, better conformation to irregular airways, epithelialization within the stent that allows for mucociliary clearance, and a greater ease of placement [16]. Granuloma formation and stent fracture have been reported around stents in benign airway obstruction with a frequency of up to 14.6% and 12.2%, respectively. It may be more common in patients with benign airway obstructions [10,16,17].

Our study demonstrated that patients with benign airway obstruction who underwent SEMS placement to relieve symptoms had worse lung function and were older compared with patients with malignant disease who underwent SEMS placement. Most of our patients with benign airway obstruction presented with dyspnoea, whereas patients with malignant airway obstruction presented with cough and/or respiratory failure. We observed a higher SEMS complication rate in patients with benign airway obstruction. Granuloma formation and SEMS fracture, in particular, were more common in patients with benign airway obstruction compared with patients with malignant disease. This was probably due to the longer period of time the stent was present in the airway and exposure of the stent to the natural environment and due to the excessive compression-decompression cycles of the airway wall during breathing and cough (stress fracture). Most complications were managed successfully and safely by experienced bronchoscopists.

At our institute, surgical treatment in patients with benign airway diseases is the first choice. If patients were unsuitable for surgical intervention because of poor lung function, comorbidities, or refusal to undergo surgery, conservative management and close monitoring are advised. SEMS placement is considered when patients present with severe symptoms that affect quality of life. The high incidence of dyspnoea at the time of presentation in patients with benign airway obstruction may be explained by the poorer lung function, older age, and increased comorbidities in these patients compared with those with malignancy.

Presenting symptoms resolved in three-fourths of the patients with benign airway obstruction and in half of the patients with malignant airway obstruction after SEMS placement. This difference is probably related to the isolated involvement of the central airway in patients with benign airway obstruction. Patients with malignant airway obstruction likely had higher rates of lung parenchymal involvement because of tumours, lymphangitic spread of malignancy, tumour emboli, and wasting syndromes associated with malignancy, decreasing improvement in presenting symptoms after SEMS placement.

SEMS fractures are not rare in patients with central airway obstruction [16], and they were overall more common in patients with benign airway obstruction. However, SEMS fracture rates were similar in patients with benign airway obstruction and in patients with malignant airway obstruction after 30 and 60 days of follow-up. Patients with malignancy-induced central airway obstruction were followed for a median of 57 days, whereas patients with benign airway obstruction were followed for 429 days. The short life expectancy in patients with malignancy may be inadequate for the development of long-term complications like granuloma formation and stent fractures. Conversely, patients with malignancy-induced central airway obstruction may develop certain complications like granuloma formations faster after SEMS placement compared with patients who have benign airway obstructions. These complications may be related to the underlying malignancies.

Douglas E. Wood reported that uncovered stents have the theoretical benefit of neo-epithelialization with incorporation of the stent into the airway wall and that this incorporation is permanent and, once the stent is seated, repositioning or removal is nearly impossible. This neo-epithelialization may be especially troubling when tumour ingrowth or granulation tissue produces recurrent obstruction inside the stent [20]. Our data do not support this observation because the stent type had been selected when placement. Most patients with benign disease in our study had airway malacia, and we selected uncovered stents. Nevertheless, there were no significant differences in complications and resolution of symptoms between patients with benign airway obstructions who underwent covered stent placement and those who underwent uncovered stent placement (Table 5).

The overall complication rates in our study are similar to those reported in previous studies [10,16,17]. SEMS-related granulation tissue formation and stenosis can be managed with a variety of flexible bronchoscopic interventions including electrocautery, cryotherapy, laser photocoagulation, radiofrequency ablation, and stent removal (when necessary). In our report, management of SEMS-related complications was feasible, and the success rate was more than 80%. However, such interventions for SEMS-related complications require experienced bronchoscopists who are familiar with techniques like electrocautery, balloon dilation, cryotherapy, and other interventional pulmonary procedures. Patients who require SEMS placement must be monitored closely for related complications.

Notwithstanding our use of the Ultraflex stents in carefully selected patients, silicon stents remain the first choice in patients with benign airway obstruction. Silicone stents with defined diameter can be repositioned and removed easily. In addition, silicon stents have little tissue reactivity, and minimal granulations form after placement. Because of the solid character of these stents, little tumour ingrowth or granulation was found after placement. Silicone stents also can be easily modified by cutting a portion of the stent to allow customization to airway anatomy [20]. In our hospital, patients with benign airway narrowing were evaluated to receive surgical treatment or silicon stent placement first.

Our study has some limitations. First, we did not perform a controlled study for airway stents; however, we did not find any obvious diversity signifying that airway stenting did not worsen the survival. Blinded, randomized, and controlled trials are hard to perform in these subjects owing to the practices. Second, SEMS placement was not recommended in patients with benign disease after the FDA warning in 2007. However, most of the SEMSs in our study were placed before 2007, and we sought to provide an outcome analysis for these subjects. We selected patients with benign disease according to poor lung function, comorbidities, and refusal to undergo surgery. The placement of an SEMS did improve most respiratory symptoms and signs in patients in this study and made further treatment possible. Finally, the factors that contribute to complications may be too complex to analyze even though our study revealed a higher rate of stent fracture and granulation formation after SEMS placement in patients with benign diseases.

## Conclusion

Surgical intervention should be the initial management option for patients who develop benign central airway obstruction and are otherwise surgical candidates. Airway prostheses including silicone stents or SEMS could be considered to treat symptoms in patients who are poor surgical candidates, at prohibitive risk for general anaesthesia, or have refused surgery. SEMSs could be placed under flexible bronchoscopy and conscious sedation with minimal immediate procedure-related complications. SEMSs are also reasonable management options for the palliation of symptomatic central airway obstruction related to malignancy. Our experience confirms that the use of SEMSs for benign severe central airway obstruction should be restricted to the treatment of severe symptoms in highly selected patients who are not surgical candidates, refuse surgery, or are at prohibitive risk for immediate complications after rigid bronchoscopy and related anaesthesia (which is required to place silicone stents). This procedure should be undertaken in these patients only after they consent to the procedure and understand the potential complications, which include serious morbidity and potential mortality. Interventional pulmonary techniques can be used with reasonable success to address some SEMS-related complications.

## List of abbreviations used

SEMSs: Self-expandable metallic stents; IRB: institutional review board; IQR: interquartile range; FDA: US Food and Drug Administration

## Competing interests

The authors declare that they have no competing interests.

## Authors' contributions

FTC and SML developed the idea for this manuscript and wrote it. FTC, HCC, and CLC performed the procedures. FTC, CTY, CHK, and HPK collected and analyzed the data. All authors read and approved the final manuscript.
